# The relationship between leaf area index and microclimate in tropical forest and oil palm plantation: Forest disturbance drives changes in microclimate

**DOI:** 10.1016/j.agrformet.2014.11.010

**Published:** 2015-02-15

**Authors:** Stephen R. Hardwick, Ralf Toumi, Marion Pfeifer, Edgar C. Turner, Reuben Nilus, Robert M. Ewers

**Affiliations:** a Department of Physics, Imperial College London, Blackett Laboratory, Prince Consort Road, London SW7 2BB, United Kingdom; b Department of Life Sciences, Imperial College London, Silwood Park Campus, Buckhurst Road, Ascot SL5 7PY, Berkshire, United Kingdom; c University Museum of Zoology Cambridge, Downing Street, Cambridge CB2 3EJ, United Kingdom; d Sabah Forestry Department, Forest Research Centre, Sepilok, PO Box 1407, 90715 Sandakan, Sabah, Malaysia

**Keywords:** Microclimate, Leaf area index, Land use change, Tropical forest, Disturbance, Oil palm

## Abstract

Land use change is a major threat to biodiversity. One mechanism by which land use change influences biodiversity and ecological processes is through changes in the local climate. Here, the relationships between leaf area index and five climate variables – air temperature, relative humidity, vapour pressure deficit, specific humidity and soil temperature – are investigated across a range of land use types in Borneo, including primary tropical forest, logged forest and oil palm plantation. Strong correlations with the leaf area index are found for the mean daily maximum air and soil temperatures, the mean daily maximum vapour pressure deficit and the mean daily minimum relative humidity. Air beneath canopies with high leaf area index is cooler and has higher relative humidity during the day. Forest microclimate is also found to be less variable for sites with higher leaf area indices. Primary forest is found to be up to 2.5 °C cooler than logged forest and up to 6.5 °C cooler than oil palm plantations. Our results indicate that leaf area index is a useful parameter for predicting the effects of vegetation upon microclimate, which could be used to make small scale climate predictions based on remotely sensed data.

## Highlights

• Microclimate was monitored in primary forest, logged forest and oil palm plantation.
• There were strong relationships between leaf area index and diurnal climate.
• Logged forest is up to 2.5 °C warmer on average than primary forest.
• Oil palm plantations are up to 6.5 °C warmer on average than primary forest.
• Forest disturbance led to desiccation of the air near the forest floor.

## 1 Introduction

Microclimate influences a wide range of important ecological processes, such as plant growth and soil nutrient cycling (Bonan, 2008). Species can exploit fine-scale variations in climate (Suggitt et al., 2012) and models that incorporate these microclimatic variations are better at predicting population dynamics (Bennie et al., 2013). However, outputs from global and regional climate models generally have horizontal resolutions of 10–100 km, although higher resolution climate projections are becoming available (Platts et al., 2014). This mismatch between what influences organisms and what can be measured could potentially lead to inaccuracies when considering the ecological impacts of a changing climate. This is especially important in the habitat-heterogeneous tropical zone, where climate change effects may be felt earlier and where the impacts on biodiversity are likely to be large (Deutsch et al., 2008, Tewksbury et al., 2008). Fine-scale heterogeneity in the future climate may provide microrefugia of tolerable climate that will help species to persist (Noss, 2001). Conversely, heterogeneity in present-day microclimate that is unaccounted for in models may mean that species have greater climatic tolerances than is currently thought (Logan et al., 2013).

Variability in climate on the micro-scale is driven by topography and vegetation cover. Topographical climate variations include effects due to elevation, cold air drainage, wind exposure, slope and aspect (Dobrowski, 2010). These physical processes are relatively well understood; however, running regional climate models at high enough resolutions to accurately simulate these effects is computationally expensive. Vegetation has long been known to modify the climate near the ground (Geiger, 1950). Recent work has shown that climate differences between different habitats can be on the same scale or larger than those projected to occur under climate change (Suggitt et al., 2011) and that canopy cover has a strong influence upon extreme climate conditions (Ashcroft and Gollan, 2012). In Southeast Asia, oil palm plantations have been measured to be 2.8 °C hotter and significantly less humid than nearby forest during the day (Luskin and Potts, 2011). However, a full understanding of the relationship between vegetation and microclimate is currently lacking, but is crucial for the development of microclimate models across heterogeneous landscapes.

There are three mechanistic reasons to expect strong relationships between vegetation structure and microclimate. First, plant canopies absorb, scatter and reflect incoming solar radiation, thus reducing the amount of energy that penetrates through to the soil and below-canopy air. The amount of solar radiation absorbed by a plant canopy depends on its leaf area index (LAI), here defined as one half of the total leaf surface area projected on the local horizontal datum (Chen and Black, 1992). Dense canopies, with high LAIs, can block over 95% of visible light from reaching the Earth's surface (Bonan, 2008), and this should keep the air and soil beneath the canopy cool during the day. In temperate forests, this effect plays a major role in protecting temperature sensitive species from the impacts of climate change (De Frenne et al., 2013). Second, plant canopies absorb momentum from the air and thus wind speed decreases with depth within the canopy (Garratt, 1992). Turbulent mixing of air is therefore suppressed by vegetation and denser canopies allow less mixing than sparse canopies. As the air at the top of the canopy heats up during the day, turbulent mixing acts to force some of this hot air down towards the ground, increasing the air temperature near the ground. Therefore, this effect acts in the same direction as the absorption of sunlight: a denser canopy should result in cooler air beneath the canopy. Finally, the amount of water vapour that air can hold is strongly dependent upon the air temperature. Therefore, in two environments with the same specific humidity (mass of water vapour per unit mass of air) but with different air temperatures, the hotter environment will have a lower relative humidity than the cooler environment. Additionally, transpiration within the forest will help to keep the air moist. In line with these expectations, previous studies have shown that air within a forest canopy has a higher relative humidity than air in nearby open areas (Chen et al., 1993, Williams-Linera et al., 1998).

LAI is a physical metric of vegetation structure that is relevant to the microclimatic processes described above. LAI is commonly measured in the field, using either destructive sampling or optical techniques such as hemispherical photography (Chen et al., 1997). In recent years, biophysical products providing continuous surfaces of LAI estimates derived from remotely sensed observations such as airborne lidar (Zhao and Popescu, 2009) or the MODIS Aqua and Terra satellites have been developed (Myneni et al., 2002). If strong patterns linking LAI to microclimate can be established then there is scope for downscaling of coarse resolution climate predictions based upon remotely measured LAI data, without microclimate data needing to be physically measured at a site. This could be extremely useful in developing high resolution climate models over a large domain.

Land use is an important modifier of LAI in forests and woody biomes (Aragão et al., 2005, Pfeifer et al., 2014). Here, we investigate the relationship between vegetation cover as described by LAI and microclimate across a range of habitats in Borneo, SE Asia. We take advantage of a pre-existing mosaic of LAI caused by a history of logging, deforestation and conversion to oil palm plantation (Ewers et al., 2011).

## 2 Methods

Data were collected at Kalabakan Forest Reserve (4°33′N, 117°16′E) and Maliau Basin Conservation Area (4°49′N, 116°54′E) as part of the Stability of Altered Forest Ecosystems (SAFE) Project (Ewers et al., 2011). The Kalabakan Forest Reserve has undergone multiple rounds of selective logging since 1978 and has a highly heterogeneous landscape. The habitats in the area range from open grasslands and scrub vegetation through to closed canopy forest. Some parts of the reserve have been converted to oil palm plantation, while other sections are currently in the process of being converted. By contrast, the Maliau Basin Conservation Area receives a high level of protection and as such contains large areas of primary forest that have never been logged.

**Fig. 1:**
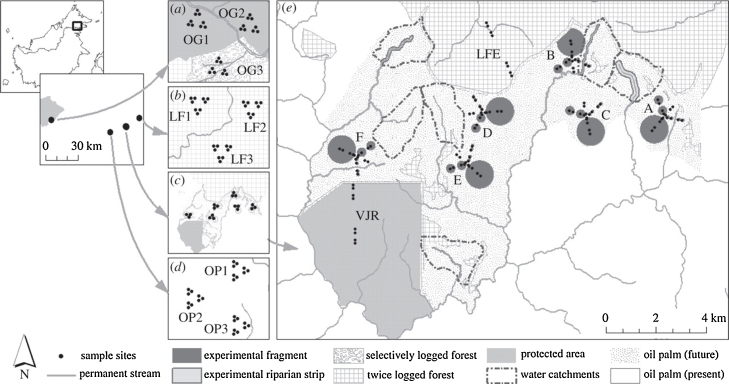
Map of the SAFE project site, showing the locations of second-order sampling points. Reproduced with permission from Ewers et al. (2011).

We used a fractal sampling design to examine spatial variation in microclimate (Ewers et al., 2011, Marsh and Ewers, 2013) with up to 579 sampling points distributed between 17 sampling blocks (Fig. 1). Microclimate sensors were located at the vertices of an equilateral triangle with edges of 56 m (1st order sample sites), with that pattern repeated at distances of 10^2.25^ m (2nd order) and again at 10^2.75^ m (3rd order). All 3rd order sites were nested within 17 sampling blocks separated by >1 km. The sampling points were located across a gradient of land use that can be broadly split into three categories; old growth forest that has never been logged (OG), old growth forest that has been logged (LF), and oil palm plantations growing on previously forested land (OP). Sites were placed to minimise variation in altitude, with the mean altitude of all sample sites being 450 m (median = 460 m; interquartile range 72 m) (Ewers et al., 2011).

Above-ground climate variables were monitored using Hygrochron iButtons (Maxim Integrated Systems, temperature accuracy <±0.5 °C, RH accuracy <±5%) suspended at a height of 1.5 m at each of the first-order sampling points (*N* = 247). The sensors were shaded from direct solar radiation due to the presence of tall vegetation at all sites. Soil temperature data were collected using Thermochron iButtons (Maxim Integrated Systems, temperature accuracy <±0.5 °C) buried at a depth of 10 cm at each of the second-order sampling points (*N* = 140). All sensors were set to record instantaneous values of climate variables every 3 h, starting at midnight each day. Above ground climate data were collected over 242 consecutive days from 15/09/2011 to 13/05/2012, while soil temperature data were collected over 189 days from 26/10/11 to 01/05/2012.

Vertical climate profile data was collected using Hygrochron iButtons, with sensors placed at heights of 0.5, 1, 2, 5, 10, 15 and 20 m above the ground. As some sensors in this study were located at the top of the canopy, all sensors were suspended beneath shallow polystyrene lids, covered on their skyward side with aluminium foil, to shield them from direct solar radiation. Vertical profiles were collected at 10 locations along a 200 m transect in logged forest. Sensors were set to record the instantaneous air temperature and relative humidity every 3 h starting at midnight each day over a period of 128 consecutive days from 30/06/2013 to 04/11/2013.

LAI data was collected between August 2012 and January 2013. Seasonal variation in LAI was not accounted for, as its effect is expected to be small. Malhado et al. (2009) report no significant seasonal variation in LAI in an Amazonian tropical forest. Myneni et al. (2007) found significant seasonal variation in LAI in evergreen forests in the Amazon; however, this variation was closely associated with the annual cycle of rainfall. Meteorological records from nearby Danum Valley Field Centre, show that the climate in this part of Sabah is aseasonal, with occasional dry spells that are usually associated with El Niño events (Walsh and Newbery, 1999). Recent data from Danum Valley (SEARRP: http://www.searrp.org/danum-valley/the-conservation-area/climate) show that no dry months, defined as months in which total rainfall was less than 100 mm (Walsh and Newbery, 1999), occurred between September 2011 and January 2013, the period during which our data was collected. Therefore, significant changes in LAI are unlikely to have occurred during this time.

Values for LAI across 16 sampling blocks were derived from canopy photographs taken using digital cameras equipped with hemispherical (fish-eye) lenses. At each second-order sampling site, we took 12 high-resolution images distributed within plots according to the Validation of Land European Remote Sensing Instruments (VALERI: http://w3.avignon.inra.fr/valeri/) project design. Cameras were mounted on tripods 1 m above the ground, looking vertically upward to the canopy. Photographs were taken under overcast conditions whenever possible to minimise anisotropy of the sky radiance. Hemispherical images were pre-processed by first extracting blue-channel pixel brightness values and then applying a threshold algorithm for separating sky from vegetation (Jonckheere et al., 2005). Resultant binary images were analysed using the free canopy analysis software CAN-EYE v6.3.8 (Weiss and Baret, 2010: http://www.paca.inra.fr/can_eye). For each site, we derived LAI corrected for foliage element clumping, limiting the field of view of the lens to values between 0° and 60° to avoid mixed pixels. LAI measured this way is estimated as plant area index, as is the case with other indirect measurements, as the estimate includes materials such as stems, trunks, branches, twigs and plant reproductive parts (Bréda, 2003). However, it is not possible to know if some leaves are present behind the stems, branches or trunk. Therefore, masking some parts of the plants to keep only the visible leaves is not correct and could lead to large underestimation of the actual LAI value, depending on the way leaves are grouped with the other parts of the plant. The full protocol that was followed for the collection of the LAI data is described in more detail in Pfeifer et al. (2012).

### 2.1 Data analysis

Both the relative and specific humidity, as well as the vapour pressure deficit (VPD), were examined, which are defined by

(1) $$\text{Relative}\;\text{humidity}=\frac{e}{e_{s}}\times 100\%$$

(2) $$\text{Vapour}\;\text{pressure}\;\text{deficit}=e_{s}-e$$

where *e* is the actual water vapour partial pressure and *e*_*s*_ is the saturated water vapour pressure and

(3) $$\text{Specific}\;\text{humidity}=\frac{m_{v}}{m_{v}+m_{a}}$$

where *m*_*v*_ and *m*_*a*_ are the mass of water vapour and dry air, respectively, in a given volume of air.

Values for the saturated water vapour pressure, *e*_*s*_, were calculated from measured air temperatures using the equation of Bolton (1980). From this, values for the VPD and specific humidity were calculated from the measured relative humidity and estimated values for air pressure based on the elevation of the sampling points. As changes in atmospheric pressure due to elevation differences affect the temperature and relative humidity of air (Andrews, 2010), all measurements were corrected for this effect using an independent dataset of climate variables taken along an elevation gradient within the Kalabakan Forest Reserve (unpublished data).

We calculated the mean and standard error for the climate and LAI values in each of the 16 sampling blocks, where each sampling block represents 8–16 second order sampling points and 24–48 first order sampling points (Fig. 1). This averages out some of the heterogeneity of LAI within sampling blocks and over the scale of one sampling block (∼1–1.5 km) provides a reliable estimate of the mean amount of incoming solar radiation that reaches the forest floor. Additionally, this minimises variation due to local effects of topography and horizontal advection of air.

We also calculated the daily standard deviations of the maximum and minimum of each climate variable. These are a measure of the amount of variation from day to day in each variable over the nine month study period. Larger values indicate that the variable fluctuated over a wider range of values. Daily standard deviations were calculated for each individual sampling point and then the sampling block means and standard errors were computed.

We used linear regression to examine the relationship between mean LAI and mean climate values.

## 3 Results

**Fig. 2:**
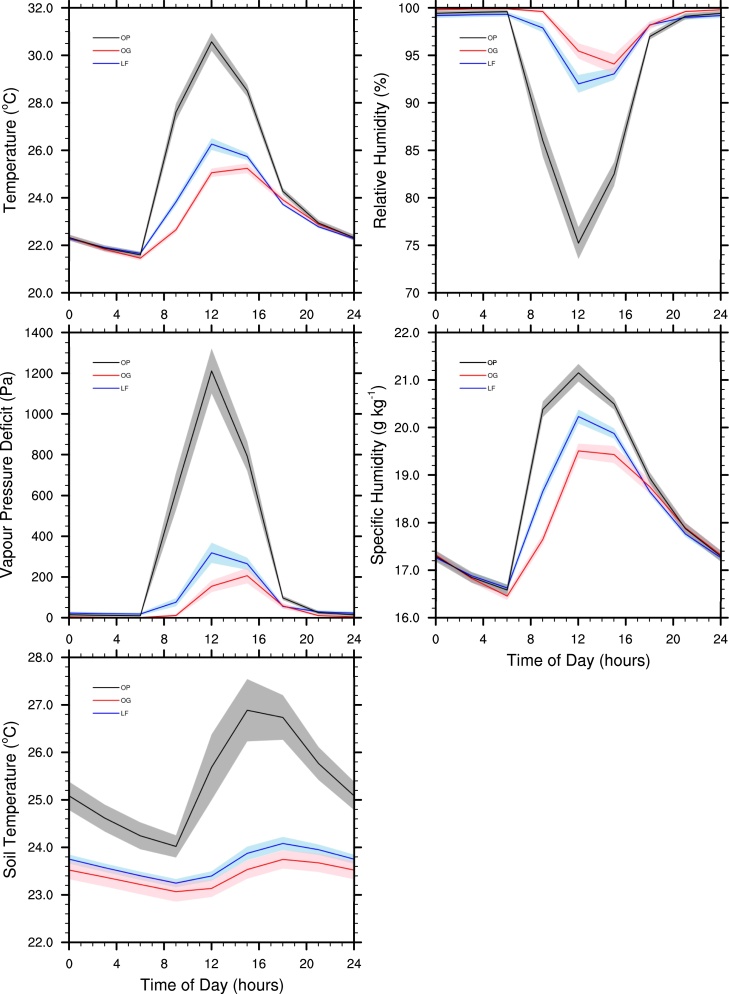
The mean diurnal cycles of the 1.5 m air temperature, relative humidity, vapour pressure deficit and specific humidity and the 10 cm soil temperature across the three land use types. Polygons show the 95% confidence intervals on the means.

The mean diurnal cycles of the five climate variables considered in this study follow similar patterns across the three land use types (Fig. 2). Air temperature reached its minimum value at 6am, before rising to its maximum value which was reached at noon in oil palm and logged forest and at 3pm in old growth forest. Air temperature then dropped off at a slower rate throughout the afternoon and continued to cool slowly overnight. The mean relative humidity was above 99% in all three land use types from 9pm until 6am. It then decreased during the morning and reached its minimum value in oil palm and logged forest at noon and in old growth forest at 3pm. The relative humidity then increased through the afternoon and evening. The mean vapour pressure deficit was small from 9pm until 6am across the three land use types. It increased during the day, reaching its maximum value at noon in oil palm and logged forest and at 3pm in old growth forest, before decreasing in the afternoon and evening. The mean specific humidity followed a similar trend to air temperature, with its minimum value occurring at 6am followed by an increase during the morning until it reached a maximum at noon in all three land use types. It then decreased throughout the afternoon and overnight. The mean soil temperature had its minimum value at 9am, and then increased during the day to its maximum value at 3pm in oil palm and at 6pm in both logged forest and old growth forest. Soil temperature then cooled overnight.

The mean maximum air temperature, soil temperature, VPD and specific humidity were largest in oil palm and smallest in primary forest, while the mean minimum relative humidity was smallest in oil palm and largest in primary forest. The mean minimum soil temperature was largest in oil palm and smallest in primary forest. Values for minimum air temperature, minimum VPD, minimum specific humidity and maximum relative humidity were very similar across the three land use types.

**Fig. 3:**
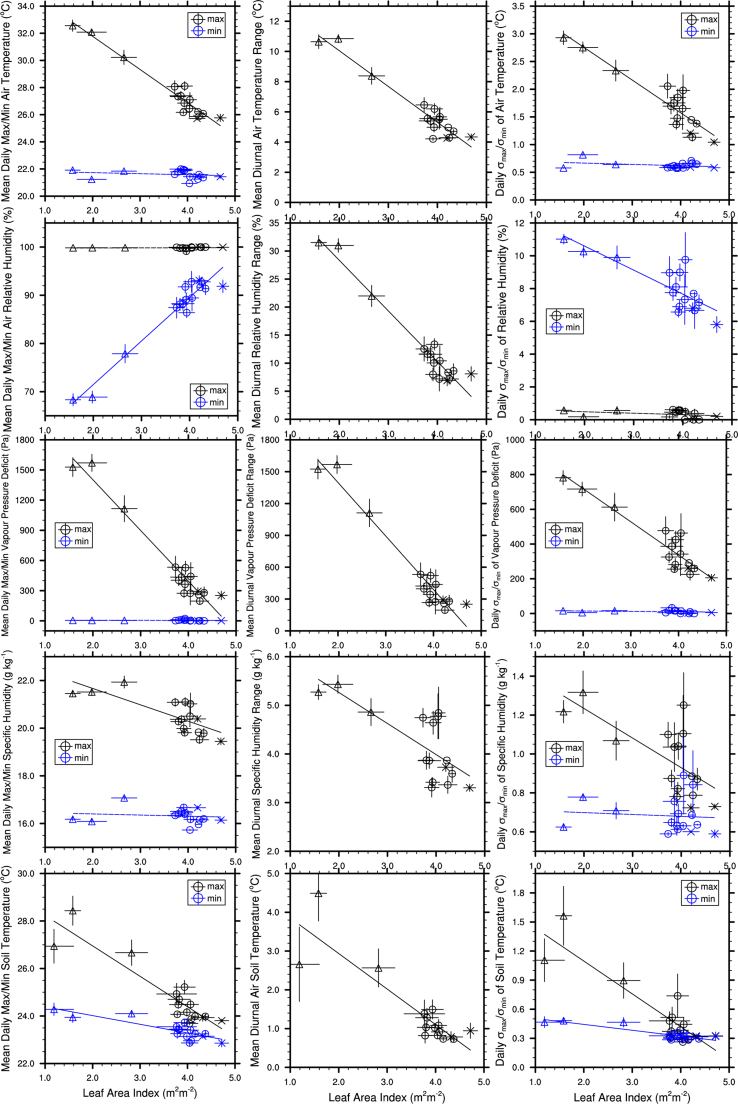
The effect of the leaf area index on the mean daily maximum and minimum (left column), diurnal range (centre column) and daily standard deviations of the maximum and minimum (right column) air temperature (first row), relative humidity (second row), vapour pressure deficit (third row) specific humidity (fourth row) and soil temperature (fifth row). Oil palm sites are marked with triangles, logged forest with circles and old growth with crosses. Dashed lines indicate non-significant trends.

Mean daily maximum air temperature (Fig. 3; *F*_1,14_ = 253, *p* < 0.001, *R*^2^ = 0.95), specific humidity (*F*_1,14_ = 20.6, *p* < 0.001, *R*^2^ = 0.60), VPD (*F*_1,14_ = 260, *p* < 0.001, *R*^2^ = 0.95) and soil temperature (*F*_1,14_ = 73.3, *p* < 0.001, *R*^2^ = 0.84) had strong negative relationships with LAI. Mean daily minimum relative humidity had a strong positive relationship with LAI (*F*_1,14_ = 245, *p* < 0.001, *R*^2^ = 0.95). The mean daily minimum soil temperature was negatively related to LAI (*F*_1,14_ = 34.7, *p* < 0.001, *R*^2^ = 0.71). There was no relationship between the mean daily minimum air temperature (*F*_1,14_ = 0.67, *p* = 0.43, *R*^2^ = 0.05), VPD (*F*_1,14_ = 0.07, *p* = 0.80, *R*^2^ = 0.005) or specific humidity (*F*_1,14_ = 0.24, *p* = 0.63, *R*^2^ = 0.02) and LAI, or between mean daily maximum relative humidity (*F*_1,14_ = 0.10, *p* = 0.75, *R*^2^ = 0.01) and LAI. The mean diurnal range of all five climate variables was negatively related to LAI (air temperature: *F*_1,14_ = 177, *p* < 0.001, *R*^2^ = 0.93; relative humidity: *F*_1,14_ = 241, *p* < 0.001, *R*^2^ = 0.95; VPD: *F*_1,14_ = 252, *p* < 0.001, *R*^2^ = 0.95; specific humidity: *F*_1,14_ = 17.6, *p* < 0.001, *R*^2^ = 0.56; soil temperature: *F*_1,14_ = 49.6, *p* < 0.001, *R*^2^ = 0.78).

The daily standard deviations of the maximum air temperature (*F*_1,14_ = 86.6, *p* < 0.001, *R*^2^ = 0.86), maximum VPD (*F*_1,14_ = 101, *p* < 0.001, *R*^2^ = 0.88), maximum specific humidity (*F*_1,14_ = 12.4, *p* = 0.003, *R*^2^ = 0.47) and minimum relative humidity (*F*_1,14_ = 29.7, *p* < 0.001, *R*^2^ = 0.68) were negatively related to LAI. The daily standard deviations of both the maximum (*F*_1,14_ = 59.7, *p* < 0.001, *R*^2^ = 0.81) and minimum soil temperature (*F*_1,14_ = 45.0, *p* < 0.001, *R*^2^ = 0.76) were negatively related to LAI. There was no significant relationship between the daily standard deviations of minimum air temperature (*F*_1,14_ = 1.58, *p* = 0.23, *R*^2^ = 0.10), minimum VPD (*F*_1,14_ = 0.65, *p* = 0.43, *R*^2^ = 0.04), minimum specific humidity (*F*_1,14_ = 0.12, *p* = 0.74, *R*^2^ = 0.01) or maximum relative humidity (*F*_1,14_ = 1.56, *p* = 0.23, *R*^2^ = 0.10) and LAI.

**Fig. 4:**
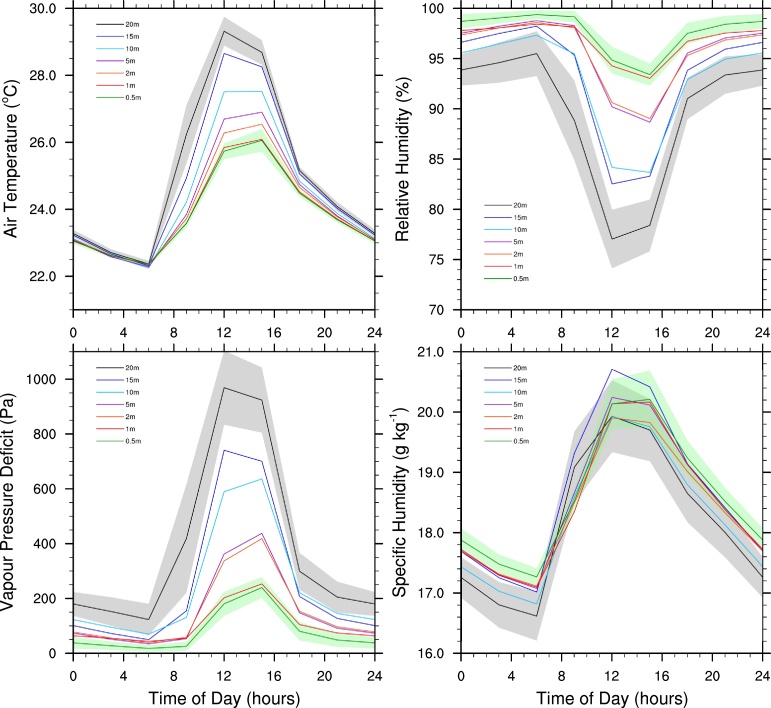
The mean diurnal cycle of air temperature, relative humidity, vapour pressure deficit and specific humidity at seven different heights above ground within a logged forest canopy. Polygons indicate the 95% confidence intervals on the means for canopy heights of 20 m and 0.5 m.

The diurnal cycles of air temperature, relative humidity and specific humidity at different heights within the forest canopy followed the same trends as detailed above for these variables 1.5 m above the ground (Fig. 4). The maximum air temperature increases with height above the ground, while the minimum relative humidity decreases. Closer to the ground the peak maximum air temperature and minimum relative humidity were reached at 3pm, while closer to the top of the canopy the peak values were reached at noon. There was a highly significant positive relationship between temperature and canopy height (*F*_1,5_ > 16.3, *p* < 0.01, *R*^2^ > 0.92) at all times of the day except at 3am (*F*_1,5_ = 6.3, *p* = 0.054, *R*^2^ = 0.56) and 6am (*F*_1,5_ = 0.088, *p* = 0.78, *R*^2^ = 0.02). Similarly, there was a highly significant negative relationship between relative humidity and canopy height (*F*_1,5_ > 16.3, *p* < 0.01, *R*^2^ > 0.80) at all times of day expect at 3am (*F*_1,5_ = 15.1, *p* = 0.012, *R*^2^ = 0.75) and 6am (*F*_1,5_ = 12.0, *p* = 0.018, *R*^2^ = 0.71), for which there were moderately significant negative relationships. There was a highly significant positive relationship between vapour pressure deficit and canopy height (*F*_1,5_ > 16.8, *p* < 0.01, *R*^2^ > 0.77) at all times of the day expect at 6am (*F*_1,5_ = 12.6, *p* = 0.02, *R*^2^ = 0.72), for which there was a moderately significant positive relationship. By contrast, there was a moderately significant negative relationship between specific humidity and canopy height during the night and early morning (midnight: *F*_1,5_ = 8.65, *p* = 0.032, *R*^2^ = 0.63; 3am: *F*_1,5_ = 11.3, *p* = 0.020, *R*^2^ = 0.69; 6am: *F*_1,5_ = 13.9, *p* = 0.014, *R*^2^ = 0.73; 9am: *F*_1,5_ = 15.8, *p* = 0.011, *R*^2^ = 0.76) but no significant relationship during the afternoon and evening (noon: *F*_1,5_ = 0.15, *p* = 0.71, *R*^2^ = 0.03; 3pm: *F*_1,5_ = 0.35, *p* = 0.58, *R*^2^ = 0.07; 6pm: *F*_1,5_ = 4.61, *p* = 0.08, *R*^2^ = 0.48; 9pm: *F*_1,5_ = 4.86, *p* = 0.08, *R*^2^ = 0.49).

## 4 Discussion

We found strong relationships between LAI and microclimate that were consistent with our hypotheses and indicate that LAI plays an important role in controlling microclimate within tropical forest ecosystems.

Simple linear regressions explain a high proportion of the variance between the climate variables studied here and the LAI. These regression formulae may be useful in microclimate modelling studies in which the effects of vegetation structure need to be parameterized. (We include the full values of the gradients and *y*-intercepts of each regression line in Table A1).

LAI is a convenient parameter as a number of standard procedures exist for the measurement of LAI, both from the ground and the air. LAI is already included in most climate and land surface models and global datasets already exist (Zhu et al., 2013), although these have not been fully validated for tropical regions to date (but see Pfeifer et al., 2014). This potentially opens an avenue to connect microclimate models to larger-scale climate models. Using the relationships we describe here, low resolution climate model output and high resolution LAI data can be combined to produce high resolution microclimate predictions.

There are other features of vegetation that could influence microclimate. One such factor is the canopy height. A higher canopy will allow less vertical mixing of warm air down to 1.5 m above the surface as there is a greater distance for turbulent eddies to penetrate. In this study, the mean canopy height was 5.3 m in oil palm plantation, 19.4 m in logged forest and 33.7 m in primary forest. Therefore, across the land use gradient considered here there is a positive correlation between the canopy height and the LAI, and as such the effects of canopy height are included in our observed trends. It is possible that some minor modifications to our trends would be necessary in situations where canopy height could play a large role and is not well correlated with LAI.

### 4.1 How LAI influences air and soil temperature

During the day, solar radiation penetrates the plant canopy and is absorbed by and heats the leaves, which in turn heat the air within the canopy. Light that is not absorbed by the canopy reaches the soil, where the majority of it is absorbed, heating the soil surface. As the soil surface warms, heat is both conducted down into deeper soil layers and is transferred into the air immediately above the soil. Therefore, the air temperature 1.5 m above the ground will be strongly affected by the amount of sunlight that is able to penetrate to that level. Sparser canopies with lower LAIs allow more light to reach the soil surface and so the 1.5 m air temperature increases more rapidly and reaches a higher maximum value than in forests with denser canopies.

The air temperature increased more rapidly and reached a higher maximum value at higher vertical levels within the canopy than near the surface. This is because more sunlight is absorbed near the top of the canopy which results in an increase in local plant and air temperature, establishing an air temperature gradient. Thus, when vertical mixing of air occurs, driven by turbulence at the top of the canopy, it acts to warm the near-surface air by forcing hotter air from the higher levels of the canopy down to lower canopy heights. Canopies with high LAIs absorb more momentum and therefore allow less vertical mixing of air within the canopy (Raupach et al., 1996), and thus act to keep the near-surface air cool. Canopies with lower LAIs allow more turbulent mixing of air within the canopy sublayer and this results in increased temperatures near the ground. In canopies with extremely low LAIs, vertical profiles such as those shown in Fig. 4 may break down as a high proportion of sunlight is able to reach the soil surface. This may result in the temperature gradient being neutralized or even inverted, in which case convective vertical mixing would act to transfer heat upwards and away from the surface.

These two processes combined probably explain the observed relationship between mean daily maximum 1.5 m air temperature and the LAI. From our dataset it is not possible to estimate the relative contributions of the two processes and this topic will be the subject of future modelling studies.

From early afternoon, air temperatures started to decrease, with the incoming solar energy being less than the flux of energy from the canopy into the free atmosphere. Additionally, convective rain showers frequently occur at this time, even during the dry season, and these could act to reduce air temperatures. During the night, the forest cools via emission of longwave radiation and air movement is dominated by buoyancy forces (Mahrt et al., 2001, Goulden et al., 2006). Drainage flows along sloped topography mean that elevation becomes the key determining factor in air temperature and, therefore, across the LAI gradient the night time temperatures are similar and there is no significant trend in minimum temperature.

The soil surface is heated by incoming solar radiation that penetrates the full depth of the plant canopy. Heat is then conducted down into the lower soil layers, however, this process takes time and so the 10 cm soil temperature lags behind the 1.5 m air temperature. The maximum temperature was probably higher beneath sparser canopies because more solar energy reaches the soil surface. There was also a significant negative relationship between minimum temperature and LAI. This was likely because the soil at 10 cm does not have enough time to fully cool down and reach equilibrium with the air temperature overnight, due its high heat capacity and relatively low thermal conductivity (Bonan, 2008).

### 4.2 How LAI influences VPD and humidity

The 1.5 m VPD was lower during the day in forests with denser canopies. The VPD is an ecologically important quantity as it has been shown to have a close relationship with transpiration rates in tropical forests (Granier et al., 1996, Motzer et al., 2005). During the day, as the temperature rises, the saturated water vapour pressure, *e*_*s*_, increases, driving an increase in the VPD and a decrease in the relative humidity. Under low LAI canopies, where the daytime temperature is higher, the VPD is also larger, while the relative humidity is lower. As the VPD increases, evapotranspiration also increases as the air has an increased capacity to hold water vapour, creating a larger potential gradient across the leaf-air and soil-air boundaries (Garratt, 1992). This increased transpiration in low LAI canopies is probably the cause of the observed trend in specific humidity, with high LAI canopies having a smaller specific humidity than low LAI canopies. This finding is similar to that reported by Law et al. (2001) from observations and models of temperate forests.

### 4.3 How LAI influences climatic variability

The daily standard deviation was greater for all five studied climate variables under canopies with lower LAIs. In other words, there is greater day to day variability in the climate near the ground for low LAI canopies than there is for high LAI canopies. This variability is probably driven by the amount of solar radiation that is incident at the top of the canopy, which will depend upon cloud cover. Canopies with high LAIs absorb the vast majority of all incident sunlight, so the amount of sunlight reaching the ground will be similar on cloudy and sunny days. By contrast, as low LAI canopies absorb a much lower fraction of the incident solar radiation, the absolute difference in the amount of sunlight that penetrates the canopy and reaches the ground on cloudy and sunny days will be quite large. Thus we would expect a greater day-to-day variability in the air and soil temperatures under low LAI canopies as was observed. As described above, the VPD and the relative and specific humidity follow the air temperature and so a greater daily variability in the air temperature leads to greater daily variability in these quantities as well.

### 4.4 Land use change, microclimate and biodiversity

Human modification of forests results in a change in climate within the forest. We found there was an increase in mean daily maximum air temperature of 2.45 °C for every decrease of 1 m^2^ m^−2^ in LAI (Table A1). In terms of land use change, this corresponds to mean maximum temperatures that are up to 2.5 °C higher in logged forest and up to 6.5 °C higher in oil palm plantations when compared to primary forest. We also found similarly large differences in air moisture content and soil temperature. Conversion of forest to oil palm is a major driver of biodiversity loss in Southeast Asia (Foster et al., 2011), and these dramatic and relatively sudden microclimatic changes are likely to be an important factor in this. Such microclimate-induced changes in biodiversity as well as direct impacts of temperature on processes such as litter decomposition are also likely to influence ecosystem functioning in modified forests. However, we are currently far from having a full understanding of how ecosystems respond to shifts in microclimate, and this is an area in need of further experimental and modelling studies.

## 5 Conclusion

We found a strong link between leaf area index (LAI) and five ecologically important climate variables in a tropical forest. This offers great potential for improved modelling of microclimate in heterogeneous landscapes, as LAI is already widely used in climate and land surface models and can be measured locally and remotely following standard methods. Human disturbance of forest systems leads to significant changes in LAI that alter the microclimate and these changes will likely have knock-on effects upon ecosystem functioning.

## Figures and Tables

**Table A1:** Values of the gradient, *m*, and *y* intercept, *c*, for the regression lines in Fig. 3, following the straight line formula, *y* = *m* × LAI + *c*, where *y* is the parameter of interest. Standard errors are included in parentheses.

Climate variable		Mean daily maximum	Mean daily minimum	Diurnal range	Daily *σ*_max_	Daily *σ*_min_
Air temperature (°C)	*m*	−2.45 (0.15)	−0.08 (0.10)	−2.37 (0.18)	−0.59 (0.06)	−0.02 (0.02)
	*c*	36.7 (0.56)	21.9 (0.37)	14.8 (0.67)	3.94 (0.22)	0.71 (0.08)

Relative humidity (%)	*m*	−0.02 (0.06)	9.05 (0.58)	−9.03 (0.58)	−0.09 (0.07)	−1.47 (0.27)
	*c*	99.8 (0.22)	53.4 (2.1)	46.4 (2.1)	0.66 (0.26)	13.5 (1.0)

Vapour pressure deficit (Pa)	*m*	−504 (31)	−0.47 (1.8)	−504 (32)	−191 (19)	−2.18 (2.7)
	*c*	2412 (116)	6.42 (6.7)	2406 (118)	1102 (70)	18.7 (10)

Specific humidity (g kg^−1^)	*m*	−0.69 (0.15)	−0.05 (0.10)	−0.64 (0.15)	−0.15 (0.04)	−0.01 (0.03)
	*c*	23.0 (0.56)	16.5 (0.37)	6.54 (0.56)	1.54 (0.15)	0.72 (0.11)

Soil temperature (°C)	*m*	−1.28 (0.15)	−0.37 (0.06)	−0.92 (0.13)	−0.34 (0.04)	−0.06 (0.01)
	*c*	29.5 (0.56)	24.7 (0.22)	4.77 (0.48)	1.77 (0.15)	0.56 (0.04)
